# Differentiation of Human Epidermal Neural Crest Stem Cells (hEPI-NCSC) into Virtually Homogenous Populations of Dopaminergic Neurons

**DOI:** 10.1007/s12015-013-9493-9

**Published:** 2014-01-08

**Authors:** Alla Narytnyk, Bernard Verdon, Andrew Loughney, Michele Sweeney, Oliver Clewes, Michael J. Taggart, Maya Sieber-Blum

**Affiliations:** 1 Institute of Genetic Medicine, Newcastle University, Centre for Life, Central Parkway, Newcastle upon Tyne, NE1 3BZ UK; 2 Maternity Unit, Women’s Services, Newcastle upon Tyne Hospitals NHS Foundation Trust, Royal Victoria Infirmary, Queen Victoria Road, Newcastle upon Tyne, NE1 4EP UK; 3 Institute of Cellular Medicine, Newcastle University Medical School, Framlington Place, Newcastle upon Tyne, NE2 4HH UK

**Keywords:** Stem cell, Somatic stem cell, Adult stem cell, hEPI-NCSC, Neural crest, Dopaminergic neuron, NURR1, FOXA2, SB431542, IWP-4, CHIR99021

## Abstract

Here we provide a protocol for the directed differentiation of hEPI-NCSC into midbrain dopaminergic neurons, which degenerate in Parkinson’s disease. hEPI-NCSC are neural crest-derived multipotent stem cells that persist into adulthood in the bulge of hair follicles. The experimental design is distinctly different from conventional protocols for embryonic stem cells and induced pluripotent stem (iPS) cells. It includes pre-differentiation of the multipotent hEPI-NCSC into neural stem cell-like cells, followed by ventralizing, patterning, continued exposure to the TGFβ receptor inhibitor, SB431542, and at later stages of differentiation the presence of the WNT inhibitor, IWP-4. All cells expressed A9 midbrain dopaminergic neuron progenitor markers with gene expression levels comparable to those in normal human substantia nigra. The current study shows for the first time that virtually homogeneous populations of dopaminergic neurons can be derived ex vivo from somatic stem cells without the need for purification, with useful timeliness and high efficacy. This novel development is an important first step towards the establishment of fully functional dopaminergic neurons from an ontologically relevant stem cell type, hEPI-NCSC.

**Electronic supplementary material:**

The online version of this article (doi:10.1007/s12015-013-9493-9) contains supplementary material, which is available to authorized users.

## Introduction

Generating large numbers of neurons for disease modeling, drug discovery and future cell replacement therapy in Parkinson’s disease is an active and promising field of research in which much progress has been achieved in recent years. Chambers et al. [1] showed that dual SMAD inhibition leads to complete neural conversion of human embryonic stem cells (hESC). The same group built on this observation and generated a cell population of which 60–80 % expressed the neuronal marker genes NURR1, LMX1a, FOXA2 and tyrosine hydroxylase (TH) by day 50 in culture [2]. The authors did not, however, show that the cells also expressed the key midbrain marker EN1. Subsequently, Xi et al. [3] reported a study in which populations with up to 87 % cells double positive for NURR1 and FOXA2 were obtained from human embryonic stem cell (hESC) cell lines by day 28 of culture. Approximately 20 % of total cells were triple positive for the marker genes TH, GIRK2 and EN1, and were thus identified as A9 dopaminergic neurons. Overall, current state-of-the art methods yield populations that after prolonged periods of time in culture are enriched to varying degrees with cells that show characteristics of functional dopaminergic neurons. Clearly, however, in order to furnish the above experimental and clinical aims, there remains a need to improve upon the efficiency and effectiveness of the differentiation process.

hEPI-NCSC are multipotent somatic stem cells, which are remnants of the embryonic neural crest and persist postnatally and into adulthood in the bulge of hair follicles [4–8]. By taking advantage of the migratory ability of neural crest cells, hEPI-NCSC can be isolated as a highly pure population of multipotent stem cells [5]. hEPI-NCSC can be expanded ex vivo rapidly into millions of stem cells and they can differentiate into all major neural crest derivatives [9]. In mouse models of spinal cord injury, intraspinal mouse EPI-NCSC elicited significant improvement in sensory connectivity through generating GABAergic neurons, re-myelination, neurotrophic support and modulation of scar formation [10, 11]. Embryonic neural crest cells emigrate from the dorsal aspect of the neural tube along the entire neuraxis and invade the embryo by various routes [12]. A subset of multipotent neural crest cells migrates below the ectoderm and subsequently invades the ectoderm [13] to develop into melanocytes, which give skin and hair their color, and to form a reservoir of multipotent neural crest-derived stem cells in the follicular bulge. By contrast, midbrain dopaminergic neurons are born in the ventral neural tube at the isthmus, the zone of convergence of three growth factors essential for dopaminergic neuron development, fibroblast growth factor 8 (FGF8), sonic hedgehog (SHH) and WNT proteins [14].

The rationale behind the current study was three-fold. First, active canonical WNT signaling, which is a prerequisite for midbrain dopaminergic neuron differentiation, is a hallmark of neural crest cells. Second, similar to midbrain floor plate cells, the neural crest is predestined to generate catecholaminergic neurons, as it gives rise to the autonomic and enteric nervous systems. Third, neural crest stem cells are ontologically closely related to central nervous system precursor cells, as during embryonic development a higher order stem cell generates both spinal cord progenitor cells and neural crest stem cells [15]. We therefore based the experimental design on commonalities and differences in embryonic development of neural crest cells and midbrain dopaminergic neuroblasts.

## Materials

### Equipment

- Class II safety hood
- Two-gas incubator at 5%CO_2_ and 5%O_2_
- Inverted microscope
- Dissecting microscope
- Fluorescence microscope
- Thermocycler
- Andor Revolution XD System or other means to capture calcium flux
- Illumina platform

### Reagents

**NP2 medium** consisted of D-MEM/F-12/GlutaMAX, 1x Pen/Strep, FBS (1 %); ITS+3 or SITE+3, β-mercaptoethanol (10 μM), Heparin (2 μg/ml); EGF (20 ng/ml); rhFGF2 (10 ng/ml) and NT-3 (10 ng/ml).

**Patterning and differentiation** was achieved in DMEM-F12-Glutamax medium that was supplemented with 1 % fetal bovine serum (FBS; where indicated), penicillin/streptomycin, SHH-C24II (500 ng/ml; 100 ng/ml), FGF8b (10 ng/ml), rhGDNF (5 ng/ml), rhBDNF (20 ng/ml), db c-AMP (1 mM) ascorbic acid (200 μM), and CHIR99021 (0.5 μM; where indicated), B27 (minus vitamin A) supplement, brain derived neurotrophic factor (rhBDNF, 20 ng/ml), glial cell line derived factor (GDNF, 5 ng/ml), ascorbic acid and dibutyryl cyclic AMP (db c-AMP; 100–500 μM). The WNT inhibitor, IWP-4 [ref 37] (100 nM days 4–6; 1 μM thereafter), SB431542 (10 μM) and LDN-193189 (100 nM) were added as indicated. Sources of materials are listed in Supplemental Table 1. Cells were seeded at 2,500–5,000 cells per CellStart-coated 35-mm plate. They were sub-cultured once onto poly-D-lysine (20 μg/ml), fibronectin (2 μg/ml) and laminin (1 μg/ml) coated glass coverslips at various times during differentiation and at different cell densities. Neither the timing of subculture nor the seeding density grossly affected differentiation, except that at late stages of in vitro differentiation not all cells resumed the phase bright multipolar neuronal morphology, but tended to remain flattened. Culture medium needs to be exchanged with fresh medium every other day. Sources for reagents are provided in Supplemental Table 1.

## Methods

**Primary explants** were obtained exactly as we have described previously [9]. De-identified pubic hairy skin biopsies were obtained with ethical approval (REC REF: 08/H0907/1) from consenting individuals undergoing repeat elective Caesarean sections. The cells were obtained and expanded ex vivo exactly as we have described previously [9].

**Indirect immunocytochemistry** was performed exactly as we have described previously [9]. In brief, cells on glass coverslips were fixed with 4 % paraformaldehyde (PFA), rinsed, blocked (2 % goat serum or 1 % bovine serum albumin [BSA]), primary antibodies added and incubated overnight in the cold. Cultures were then rinsed, incubated with fluorescent-conjugated secondary antibodies (1:200), rinsed and finally mounted with a slide and Vectashield-plus-DAPI (Vector Laboratories, Cat# H-1500). Sources and dilutions of antibodies are listed in Supplemental Table 1.

**Real-time polymerase chain reaction (qPCR)** was performed exactly as we have described previously [9]. Briefly, cDNA was synthesized following RT-PCR and set up for PCR reaction as follows: 15 μl SYBR Green (Sigma Cat# S4438), 12 μl ddH20, 1.5 μl cDNA, 1.5 μl primer set (SABiosciences), 10 μl aliquots delivered into three wells of a 384 well plate (Greiner Cat# 785290), and amplified in a Taqman 7900HT thermocycler as follows: 95 °C, 10 min followed by 40 cycles of 95 °C (15 s), 60 °C (60 s) and 70 °C (30 s), followed by melting curve analysis to confirm single product amplification. For analysis the average of the Ct triplicates for the PCR product of each gene was determined, normalized against the average of three housekeeping genes (HKG), glyceraldehyde 3-phosphate dehydrogenase (GAPDH), TATA box binding protein (TBP), succinate dehydrogenase complex subunit A (SDHA) and expressed as a percentage of the average of the three HKG. Primer pairs are listed in Supplemental Table 1.

### Human Substantia Nigra

Frozen midbrain cryo-sections (15 μm) of human substantia nigra from three healthy donors were obtained from Parkinson’s UK Brain Bank, Centre for Neuroscience, Imperial College, London. The readily identifiable dark substantia nigra was microdissected under brightfield optics, the tissue collected, pooled, dissolved in TRIzol and subsequently processed for real-time PCR.

### Calcium Imaging

Changes in [Ca^2+^]_i_ were assessed using the calcium-sensitive dye Fluo-4. Cells grown in μ-Slides (IBIDI cat#80821) were loaded with 1 μM Fluo-4 AM and 0.02 % Pluronic F-127 in culture medium for 35 mins at 37 °C and maintained on the stage of a temperature-controlled (36.8 °C) inverted Olympus IX81 microscope. The Fluo-4-loaded cells were washed twice with HEPES buffer (NaCl 145 mM; MgSO_4_ 1 mM; KCl 5 mM; glucose 10 mM; CaCl_2_ 2.5 mM; HEPES 10 mM; pH 7) and then allowed to equilibrate for 20 min. Fluo-4 fluorescence images (488 nm excitation, 525 nm emission, 50 nm long-pass filter) were collected and recorded using an Andor Revolution XD System with Andor IQ software version 1 (Belfast, UK). Images were acquired at 12–25 frames per sec for up to 2 mins. Real-time movie files of continuously recorded data were viewed to assess changes in cellular fluorescence occurring spontaneously or in response to agonist stimulation. The number of cells exhibiting calcium fluctuations were noted as percentage of all cells in the field of view. After equilibration, activity was recorded in HEPES buffer (spontaneous activity) and following the addition to the dish of either 300 μM ATP, 30 μM acetylcholine, 10 μM phenylephrine, 50 μM L-glutamate or 80 mM KCl (HEPES buffer with iso-osmotic substitution of NaCl for 80 mM KCl). After recording responses, cells were washed with HEPES buffer and left to rest for 15 min before addition of another reagent. At the end of each experiment the response to addition of 10 μM cyclopiazonic acid was recorded to demonstrate Ca^2+^ release from intracellular stores. Data were analyzed using ImageJ (http://rSB431542web.nih.gov/ij/) software.

### Gene Expression Profiling

RNA was isolated from cells grown (a) in expansion medium for 7 days, and (b) subsequently for 7 days in NP medium exactly as we have described previously [9] and above. Gene expression profiling was performed by the Genome Centre, University of London, London EC1M 6BQ, on a Human HT-12 v4 array using the Illumina platform. The two profiles have been deposited in the Gene Expression Omnibus (GEO); GEO accession number GSE42678; http://www.ncbi.nlm.nih.gov/geo/.

## Notes

1. Pre-differentiation of the multipotent hEPI-NCC into neural stem cell-like cells is essential in our hands. Based on morphology and early neuronal marker expression, ex vivo expanded hEPI-NCSC did not differentiate efficiently into neuroblasts. We thus rationalized that the multipotent stem cells first have to be pre-differentiated into neural stem cell-like cells. Based on our previous work [16–19] and that of others [20–22] we developed an empirical culture medium, termed neural progenitor 2 (NP2) medium. After pre-treatment with NP2 medium, the originally stellate cells [9; Fig. 1A] changed their morphology to elongated cells with short processes and they continued to proliferate (Fig. 1B). Results from pilot experiments (not shown) showed that minimum required exposure to NP2 medium was 24 h but could be extended to up to 6–7 days.

**Fig. 1:**
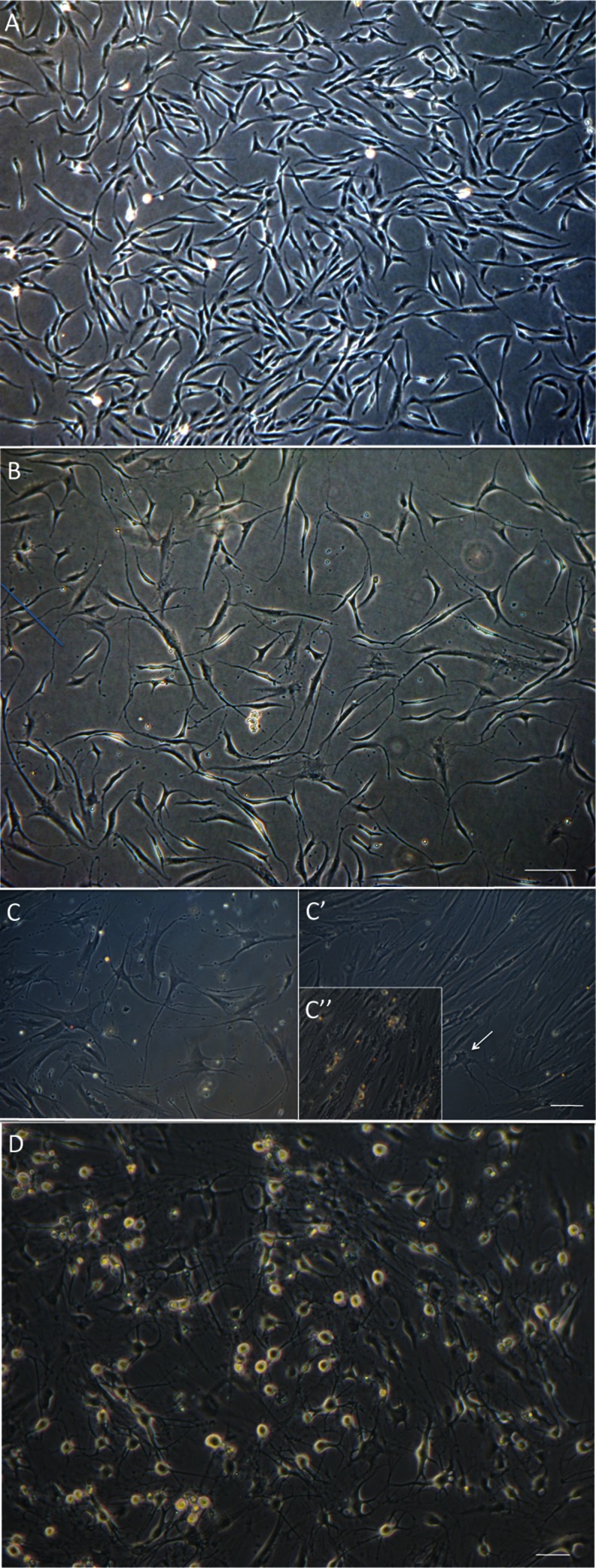
Phase contrast images of differentiating hEPI-NCSC. **a** hEPI-NCSC with stellate morphology in expansion culture prior to differentiation. **b** By differentiation day 4, virtually all cells were bipolar with short processes. Bar, 100 μm. **c** In the presence of IWP-4 virtually all cells were multipolar with long processes and dopaminergic neuron morphology. In contrast, in the absence of IWP-4, the majority of cells often, but not always, flattened and lost neuronal morphology by day 14–18 (C’). Many neurons appeared unhealthy (e.g., *arrow*) and subsequently died. In subsequent days there were areas with widespread cell death (C”). Bar, 100 μm. **d** By day 25–30 differentiated cells had assumed mature multipolar neuronal morphology with phase-bright somata. Bar, 50 μm
2. Inhibition of WNT signaling is important for long-term maintenance of the neuronal phenotype. In the absence of the WNT inhibitor, IWP-4, cells often, but not always, started to lose neuronal morphology around day 14 of differentiation and cells with neuronal morphology died (Fig. 1C’, C”). In contrast, in the presence of increasing doses of IWP-4, neuronal morphology and cell viability were maintained (Fig. 1C). In addition, the continued presence of SB431542 was required for maintaining neuronal morphology. Data from pilot experiments (not shown) suggested that differentiation progressed in the absence of CHIR99021. Exposure of the cells to a low dose (0.5 μM) of CHIR99021 for 24 h, however, seemed to accelerate/stabilize OTX2 expression. This low dose of CHIR99021 was therefore routinely added to ensure reproducibility. When the neurotrophins BDNF and GDNF were added more than 4 days after addition of SHH (500 nM) and FGF8, all cells died (data not shown). To ensure reproducibility, neurotrophins were therefore added at the onset of differentiation. By day 25–29, cells had acquired midbrain dopamine neuron morphology (Fig. 1D). The experimental design is summarized in Fig. 2. By day 5, the large majority of cells were immunoreactive for OTX2, NURR1, FOXA1, TH and β-III tubulin. All cells expressed immunoreactivity for OTX2, β-III tubulin and perinuclear TH. Subsets of 89.2 ± 17.4 % of cells expressed FOXA2 and 93.1 ± 11.1 % of cells expressed NURR1. Punctate TH immunoreactivity was located in perinuclear areas but not yet in distal processes (Fig. 3; Table 1). By day 25, cells showed distinct neuronal morphology. All cells were intensely immunopositive for the midbrain marker genes, dopamine (Fig. 4a), vesicular monoamine transporter-2 (VMAT2) (Fig. 4b), EN1 (Fig. 4c), β-III tubulin (Fig. 4d), NURR1 (Fig. 4e, E”), LMX1b (Fig. 4f), GIRK2 (Fig. 4g), PITX3 (Fig. 4h), FOXA2 (Fig. 4i, I’), synaptophysin (Fig. 4j) and TH (Fig. 4k, l, m) (Table 1). FOXA2 immunoreactivity was expressed by 89.6 % of cells (Table 1). Punctate TH immunoreactivity was localized both in the somata and processes. Immunoreactivity for 5-HT (Fig. 4E’) was at background levels. Nestin immunofluorescence (Fig. 4J’) was also detectable at background levels only. Cells maintained their neuronal morphology for a prolonged period of time, as shown in Fig. 5. Quantitative PCR confirmed expression of relevant genes at the RNA level (Table 2). Comparison to cells isolated from human substantia nigra demonstrated that expression levels were comparable to those in human midbrain dopaminergic neurons in vivo (Table 2). Whereas all scored cells expressed the dopaminergic neuron markers detailed above, 5.15 ± 7.6 % of cells also expressed KROX20. KROX20 is a hindbrain marker [23] and also a Schwann cell marker [24]. This observation indicates that a minority of cells co-expressed either a hindbrain or glia trait.

**Fig. 2:**
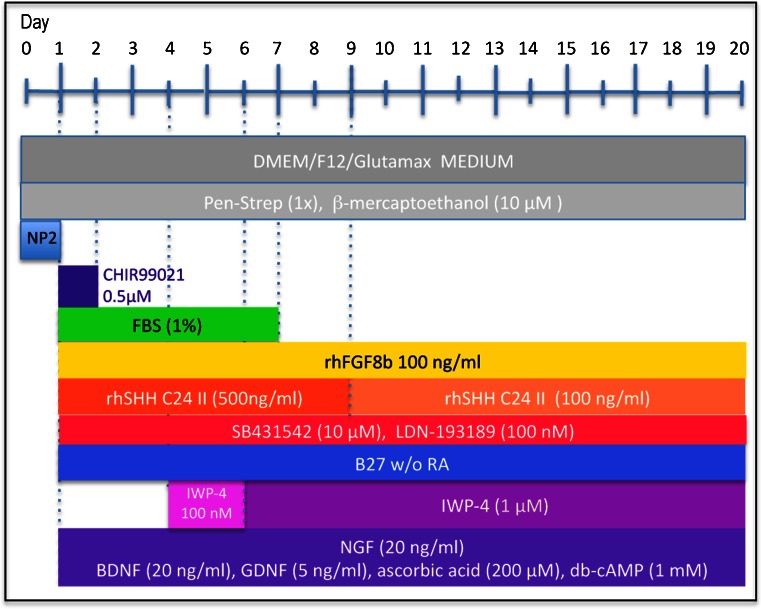
Experimental design. Cells from primary explants were expanded ex vivo for 7 days exactly as we have described previously ^[9]^, cryopreserved and subsequently used for differentiation experiments. Cells were seeded at 2,500–5,000 cells per 35 mm plate and cultured in NP2 medium for up to 6 days in a humidified atmosphere at 5 % CO_2_ and 5 % O_2_. We determined, however, that 24 h in NP2 medium is the minimal requirement. After 24 h in NP2 medium, FGF8, rhSHH C24II (500 ng/ml), LDN-193189, SB431542, B27 without retinoic acid, NGF, BDNF, GDNF, ascorbic acid and db-cAMP were added, as well as CHIR99021 (0.5 μM). The latter was removed after 24 h. At day 4, IWP-4 (100 nM), was added. At day 6, the IWP-4 dose was increased to 1 μM. At various times thereafter cells were sub-cultured using TrypLE and re-seeded onto poly-lysine and laminin-coated 13 mm glass coverslips at 6,000 cells per cm^2^ and culturing continued for various periods of time

**Fig. 3:**
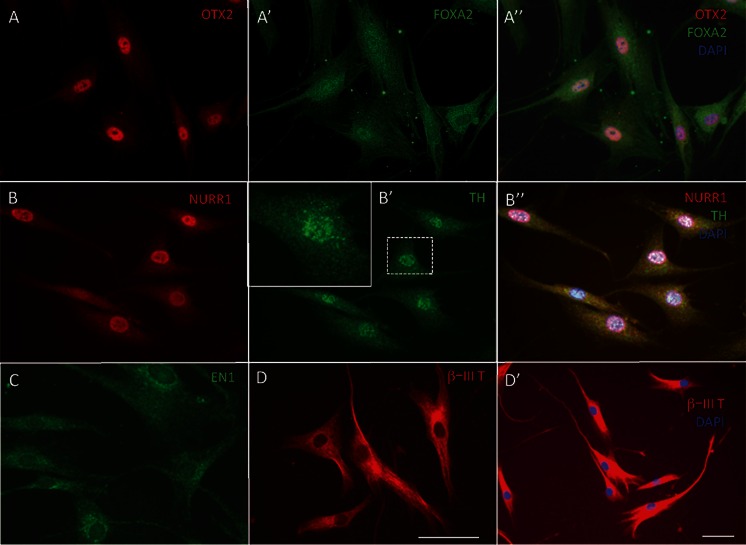
Immunocytochemistry at day 5 of culture. At day 5, all cells expressed OTX2 (**a**) in the nucleus. FOXA2 (A’, A”) merged images with DAPI nuclear stain. FOXA2 was expressed in 89.2 ± 17.4 % of nuclei, but was expressed also in perinuclear areas (see also Tables 1 and 2). **b** 93.1 ± 11.1 % of cells expressed NURR1. (B’) All cells expressed TH in the cytoplasm in perinuclear areas (inset; higher magnification), but not in distal processes. (B”) Merged images with DAPI nuclear stain. **c** All cells expressed EN1 in perinuclear areas and at lower intensity also in the nucleus. (**d**, D’) All cells expressed β-III tubulin. Bar, (**a**–**d**; D’), 50 μm

**Table 1:** Immunocytochemistry

Epitope	Percent of total ±SEM
Culture day 5	
OTX2	100
FOXA2	89.2 ± 17.4
NURR1	93.1 ± 11.1
EN1	0^a^
β-III tubulin	100
TH	100
Culture day 25	
Dopamine	100
VMAT2	100
EN1	100
FOXA2	89.6 ± 19.4
β-III tubulin	100
GIRK2	100
LMX1b	100
NURR1	100
PITX3	91.6 ± 14.6
Synaptophysin	100
TH	100
Nestin	0
5-HT	0

Data expressed as average cell counts from 20 random fields of view per antibody stain; average 3–12 cells per view. In the case of 100 % positive cells, the entire culture was inspected to validate the scores

^a^Unspecific staining in the cytoplasm negatively affected imaging of low nuclear stain

**Fig. 4:**
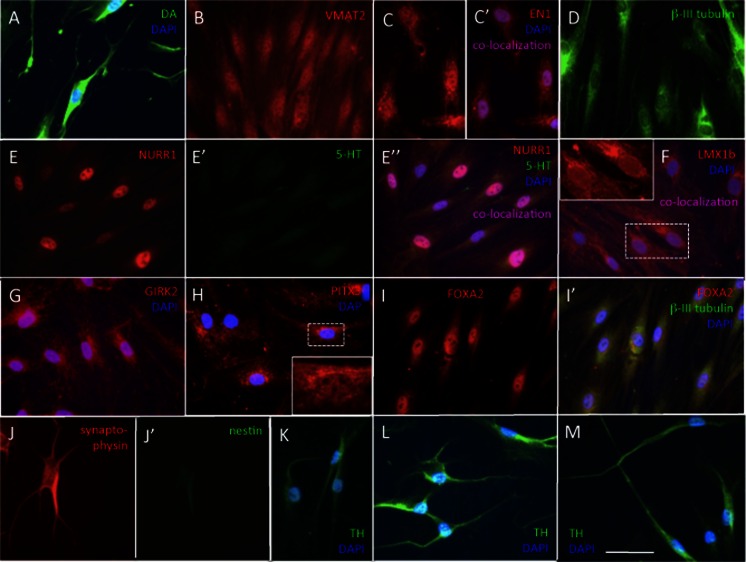
Immunocytochemistry at day 25 in culture. **a** All cells expressed dopamine (DA); neuronal morphology is evident. **b** All cells were immunoreactive for the vesicular transporter VMAT2. **c** EN1 was expressed in all nuclei (inset) and also in perinuclear areas. **d** All cells expressed the early neuronal marker, β-III tubulin. (E-E”) NURR1/serotonin double stain. All cells expressed intense nuclear NURR1 immunofluorescence (**e**, E”); (E’) background serotonin (5HT) immunoreactivity; (E”) merged E and E’ images with blue DAPI nuclear stain. **f** LMX1b was expressed in all nuclei (inset). There was also unspecific cytoplasmic fluorescence. **g** GIRK2 was expressed in all cells (GIRK2/DAPI merged image). **h** PITX3 in 91.6 ± 14.6 % of cells; there was unspecific cytoplasmic fluorescence that interfered with image acquisition; inset shows higher magnification of a nucleus. **i** FOXA2 was localized in the nucleus of 89.6 ± 19.4 % of cells. **j** Synaptophysin/nestin double stain; (J’) all cells were intensely synaptophysin immunoreactive whereas (J”) nestin immunoreactivity was at background levels. **k**, **l**, **m** All cells expressed TH in the soma and in processes. Bar, (**a**–**m**), 50 μm

**Fig. 5:**
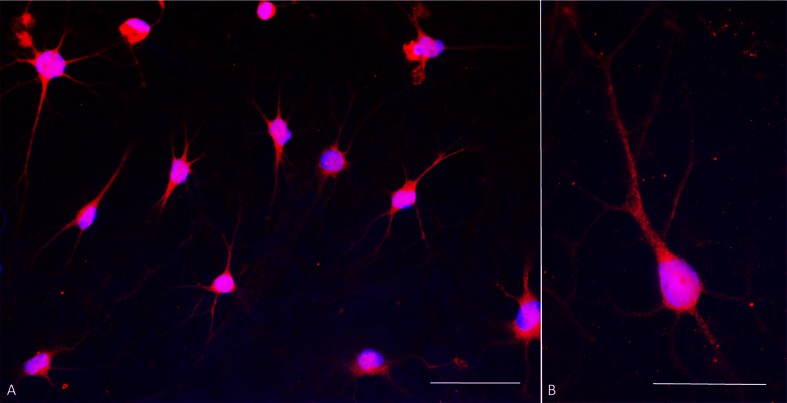
Dopamine immunocytochemistry at day 30 of culture. **a** Double stain with antibodies against dopamine (*red fluorescence*) and DAPI nuclear stain (*blue fluorescence*) shows that all cells express dopamine and have mature dopaminergic neuronal morphology. Contrast is exaggerated in order to visualize fine processes. **b** Typical dopaminergic neuron morphology at higher magnification showing punctate, presumably vesicular, dopamine immunoreactivity

**Table 2:** qPCR – comparison in vitro differentiated hEPI-NCSC with human substantia nigra

Gene	Expression level		
	(Percent average of three HKG)		
	hEPI-NCSC DA neurons	Human SN	Percent of SN
β-III tubulin	73.5 ± 0.2	91.0	80.8 ± 0.2
Tyrosine hydroxylase	73.3 ± 0.9	85.0	86.3 ± 1.0
Dopamine decarboxylase	68.6 ± 1.5	75.0	91.5 ± 2.0
Dopamine-β-hydroxylase	0	ND	–
PNMT	71.0 ± 0.7	100.4	70.7 ± 0.7
NURR1	93.3 ± 0.6	96.7	96.5 ± 0.6
PITX3	67.7 ± 0.3	79.7	84.9 ± 0.4
EN1	81.5 ± 1.6	94.4	86.3 ± 1.7
LMX1b	70.1 ± 2.7	80.9	86.6 ± 3.3
VMAT2	70.3 ± 0.4	81.8	85.7 ± 3.3
GIRK2	63.7 ± 0.7	85.2	72.7 ± 0.2
FOXA2	65.6 ± 1.4	84.4	72.1 ± 1.7
DAT	63.4 ± 1.0	73.5	86.3 ± 1.4

**Fig. 6:**
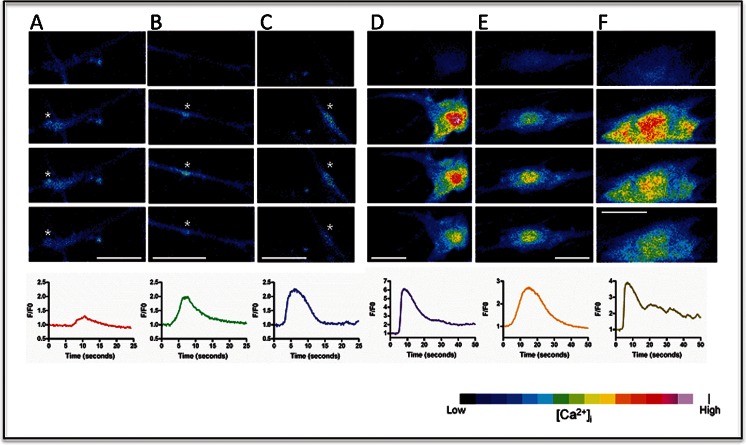
Spontaneous and agonist-mediated calcium responses. Spontaneous elevations of calcium were evident in the majority of cells and are indicated by the white asterisks of three cells depicted in panels **a**–**c**. The frames are 2.5 s apart. Purinergic-mediated calcium responses took the form of spatially propagated waves as depicted in three cells depicted in panels **d**–**f**. The frames are 2.5 s apart. The lineplots at the bottom of panels **a**–**f** illustrate the F/F0 for time periods of 25 s or 50 s for spontaneous (**a**–**c**) or agonist (**d**–**f**) changes respectively. Bars, 10 μm

*HKG* house keeping genes, *SN* adult human substantia nigra, *ND* not detected
3. As a measure of functional excitability, the intracellular calcium changes to pertinent agonists were investigated. Up to 83.2 % of cells showed spontaneous Ca^2+^ activity in the form of brief spatially restricted elevations; 80.9 % of cells also responded to cyclopiazonic acid (CPA), which empties internal calcium stores, and 77.0 % to KCl; 55.0 % of cells responded to acetylcholine, and 89.5 % to ATP (Fig. 6), 53.6 % responded to L-glutamate, and 51.0 % to phenylephrine with prolonged and spatially propagating Ca^2+^ elevations. These results suggested that subsets of cells expressed functional purinergic receptors, acetylcholine receptors, glutaminergic receptors and alpha-1 adrenergic receptors (Table 3).

**Table 3:** Calcium responses of differentiated cells

Agonist	Percent responding cells ±SEM
Spontaneous activity	83.2 ± 1.1
Acetylcholine (30 μM)	55.0 ± 7.6
ATP (300 μM)	89.5 ± 1.7
CPA (10 μM)	80.9 ± 3.8
KCl (80 mM)	77.0 ± 3.7
L-glutamate (50 μM)	53.6 ± 6.4
Phenylephrine (10 μM)	51.0 ± 5.0

*CPA* cyclopiazonic acid
4. OTX2 expression, a marker gene for successful ventralising, is essential for achieving the midbrain dopaminergic neurons phenotype. OTX2, a floor plate marker, controls the location of the isthmic organizer, which in turn defines the ventromedial dopaminergic neuron domain [14]. Expression of OTX2 was readily achieved in all hEPI-NCSC. It is conceivable that neural crest cells are highly susceptible to the SHH-mediated ventralizing signal because of their close ontological relationship with neural tube progenitor cells. However, OTX2 expression is not limited to the floor plate during embryonic development but is expressed throughout the entire cephalic mesenchyme, including in neural crest cells [25, 26]. Mutations in the OTX2 gene lead to neural crest-related craniofacial malformations [27]. OTX2 expression is thus a physiological event in the development of neural crest cells.
5. Blocking BMP and TGFβ signaling transiently with LDN-193189 and SB431542 is part of current protocols for differentiation of pluripotent stem cells into dopaminergic neurons (dual SMAD inhibition) [1–3]. In our cultures dual SMAD inhibition needed to be constant, which was likely due to the fact that hEPI-NCSC express genes for TGFβs as well as for TGFβ receptors (GEO accession number GSE42678). TGFβ signaling directs neural stem cells towards the glia cell lineage [28], which likely contributed to the unstable neuronal phenotype of hEPI-NCSC derived neuroblasts in the absence, or transient presence, of dual SMAD inhibition. Conversely, Roussa et al. [29] recognized that TGFβ3 action is essential for dopaminergic neuron differentiation. In our experiments TGFβ3 was not essential for differentiating hEPI-NCSC into midbrain dopaminergic neuroblasts, most likely because hEPI-NCSC themselves express the TGFβ3 gene endogenously (GEO accession number GSE42678).
6. Timing and dose of the GSK-3β inhibitor, CHIR99021, which is used to enhance canonical WNT signaling, were critical in order to generate FOXA2/LMX1A double positive cells from human embryonic stem cells and iPS cells [3, 30]. Thus, in culture as in the embryo, not only time windows, but also concentration ranges are critical parameters for WNT signaling to specify midbrain dopamine precursors. A short exposure of hEPI-NCSC to a low dose of CHIR99021 was not essential for dopaminergic differentiation, but was added to the experimental design to ensure reproducibility. WNT5a is expressed in NURR1+/TH+ cells in the developing midbrain [31]. WNT5a increases the portion of NURR1 positive cells that have acquired a neuronal dopaminergic phenotype [31–34]. In the present study expression of pertinent midbrain dopaminergic neuron markers was achieved in the absence of added WNT proteins and in the absence of the GSK-3β inhibitor CHIR99021 (data not shown). This may be attributed to the expression of the WNT5a gene in hEPI-NCSC (GEO GSE42678). Conversely LMX1b, which regulates WNT1 [35] may have re-activated WNT1 expression in hEPI-NCSC derived dopaminergic neurons.
7. LMX1b expression is essential for specifying midbrain identity of dopaminergic neurons [36]. Continuous WNT expression inhibits LMX1b expression. As hEPI-NCSC are highly likely to express several WNT proteins (GEO GSE42678), we added the WNT inhibitor, IWP-4 [37], in order to avoid WNT-related inhibition of LMX1b. As evidenced by cell morphology and marker expression, the presence of IWP-4 contributed to a stabilization of the neuronal phenotype and cellular health. Under these conditions serotonin immunoreactivity was consistently at background levels only.
8. Caudal to the isthmus, immediately adjacent to midbrain dopaminergic neurons lie serotonergic neurons, which later migrate dorsally within the rostral hindbrain and give rise to the Raphe nuclei [35]. LMX1b/FOXA2 double positive floor plate cells can also generate serotonergic neurons of the rostral hindbrain whereas triple positive LMX1b/FOXA2/PITX3 cells do not express serotonin [14]. WNT5a, NURR1 and GDNF up-regulate PITX3 [31, 38, 39]. The development of more than one type of neuron is a common occurrence in culture [30, 40–43]. Our current experimental design minimized this problem, as 5-HT immunoreactivity was consistently at background levels. This is likely explained by the observation that by day 25 all hEPI-NCSC-derived dopaminergic neuroblasts express the three regulatory genes, LMX1b, FOXA2 and PITX3. WNT signaling specifies midbrain—hindbrain identity in a dose-dependent manner, as CHIR99021 in doses greater than 1 μM restricted the human precursor cells to the hindbrain fate rather than the midbrain fate [3]. This result is in agreement with our observation that treatment of hEPI-NCSC with the WNT inhibitor, IWP-4, contributed to the suppression of serotonin expression. Wnt Inhibitor IWP-4 prevents palmitylation of Wnt proteins by Porcupine (Porcn), a membrane-bound O-acyltransferase, thereby blocking Wnt secretion and activity. It also blocks phosphorylation of the Lrp6 receptor and accumulation of both Dvl2 and β-catenin [37]. IWP-4 therefore also inhibits Wnt5a. For this reason it is surprising that addition of Wnt5a to up-regulate expression of PITX3 was not an essential requirement in our protocol. There are several possibilities that can explain this discrepancy. First, it is conceivable that WNT5a action was completed by culture day 6, the point in time at which IWP-4 concentration was increased to a conventionally used dose [37]. Second, it is likely that NURR1 and GDNF signalling, which also up-regulate PITX3 expression compensated for a potential lack of WNT5a. This issue could be dissected in more detail by using a WNT1-specific inhibitor, such as XAV939 [44], in place of IWP-4, as WNT5a is known to activate both the canonical and the PCP/RAC1 WNT signalling pathways in mouse dopaminergic neurons [45, 46]. Third, the sequence of events in dopaminergic neuron differentiation might be different in human neural crest-derived stem cells than in mouse pluripotent stem cells.
9. Contamination of dopaminergic neuron cultures with progenitor cells is often a problem [3]. In contrast, we found that with the current protocol there were no remaining pools of nestin positive precursor cells left in day 25 cultures of hEPI-NCSC-derived dopaminergic neurons.

## Electronic supplementary material

Below is the link to the electronic supplementary material.

Supplemental Table 1 (DOCX 110 kb)
