# Supplementary material for: Differentiation of Human Epidermal Neural Crest Stem Cells (hEPI-NCSC) into Virtually Homogenous Populations of Dopaminergic Neurons
Source: Stem Cell Rev. 2014 Jan 8;10(2):316–26. doi: 10.1007/s12015-013-9493-9 (PMC3969515; doi:10.1007/s12015-013-9493-9)
Supplement: Supplementary file 1 — (DOCX 110 kb) [file 12015_2013_9493_MOESM1_ESM.docx]

**List of sources**

ITS+3 (Sigma, Poole, UK Cat# I-2771)

GlutaMAX (Invitrogen, Cat# 35050–038)

rhFGF2 (10 ng/ml; R&D Systems, Cat# 233-FB)

rhSCF (5 ng/ml; R&D Systems, Cat# 255-SC-010/CF)

rhNT-3 (10 ng/ml; R&D Systems Cat# 267-N3-005/CF)

NeuroCult NSA (StemCell Technologies Cat# 05753)

D-MEM/F-12- Glutamax (Invitrogen, Cat# 35050–038)

β-mercaptoethanol (Sigma Aldrich #M3148; 10 µM)

Pen/Strep (Sigma Cat# P0781),

Heparin (2 µg/ml; Stem Cell Technologies #7980)

EGF (20 ng/ml; R&D Systems Cat# 236-EG)

NT-3 (10 ng/ml; R&D Systems Cat# 267-N3-005/CF)

SHH-C24II (100 – 500 ng/ml; R&D Systems Cat# 1845-SH/CF)

rhFGF-8f (R&D Systems Cat# 5027-FF/CF) (PF medium)

rhGDNF (5 ng/ml; R&D Systems, Cat# 212-GD-010/CF

rhTGF-β2 (1 ng/ml; R&D Systems, Cat# 302-B2-002/CF),

rhBDNF (20 ng/ml; R&D Systems, Cat# 248-BD-005/CF)

db c-AMP (100 µM; Sigma, Cat# D0260)

ascorbic acid (200 µM; Sigma Cat# A4544)

purmorphamine (2 µM; Stemgent)

CHIR99021 (3 µM; Stemgent)

SITE+3 (Sigma Aldrich #S5295)

rhSHH-N (200 ng/ml; R&D Systems, Cat# 1314-SH-025/CF)

B27 supplement minus Vitamin A (Gibco #12587010)

brain derived neurotrophic factor (rhBDNF, 20 ng/ml; R&D Systems, Cat#

248-BD-005/CF)

GDNF, 5 ng/ml; R&D Systems, Cat# 212-GD-010/CF

IWP-4 (Stemgent, San Diego CA)

SB431542 (10 µM; Stemgent)

LDN-193189 (100 nM; Stemgent)

fetal bovine serum (HyClone, Thermo Fisher, Cat# SH30070.02)

membrane-permeant dye Fluo-4 AM (Invitrogen, cat# F14201)

Pluronic F-127 (Invitrogen, cat# P-3000MP)

ATP (Sigma, cat# A2383)

Acetylcholine (Sigma, cat# A2661

Phenylephrine (Sigma, cat# P6126

L-Glutamate (Sigma, cat# G8415)

Cyclopiazonic acid (Sigma , cat# C1530)

1x Pen/Strep (Sigma Cat# P0781), FBS (HyClone, Thermo Fisher,

Cat# SH30070.02

Fetal bovine serum (FBS) HyClone, Thermo Fisher, Cat# SH30070.02;

rhTGF-β2 (1 ng/ml; R&D Systems, Cat# 302-B2-002/CF

rhTGF-β2 (1 ng/ml; R&D Systems, Cat# 302-B2-002/CF)

**List of Primary Antibodies**

Mouse anti- **NESTIN** (1:200) BD Biosciences Cat# 611658

Mouse anti-**βIII-TUBULIN** (1:200) Millipore/Chemicon Cat# MAB1637

Rabbit anti-**TYROSINE HYDROXYLASE** (1:500) Millipore/Chemicon Cat# AB152

Rabbit anti-**Dopa Decarboxylase** (1:200) Millipore/Chemicon Cat# AB1569

Mouse anti-**Nurr1** (1:200) Santa Cruz Cat# sc-81345

Rabbit anti-**DOPAMINE** (1:2000) Abcam Cat# ab6427

Rabbit anti-**SYNAPTOPHYSIN** (1:250) Abcam, Cat# ab32127

Rabbit anti-**FOXA2** (1:1000) Abcam, Cat#ab40874

Rabbit anti- **EN-1** (H-55) (1:200) Santa Cruz Cat#sc-66876

Goat anti-**PITX3** (N-20) (1:200) Santa Cruz Cat#sc-19307

Goat anti- **LMX1B** (N-14) (1:200) Santa Cruz Cat# sc-21231

Goat anti- **KIR3.2** **(GIRK2)** (C-20) (1:200) Santa Cruz Cat# sc-16135

Mouse anti- **VMAT2** (9E11) (1:100) Novus Biologicals Cat# NBP1-47977

Rabbit anti-**SEROTONIN (5-HT)** (1:5000) Sigma Cat#S 5545

Mouse anti-**OTX2** (1:400) Abcam Cat #ab130238

**List of Secondary antibodies**

|  |  |  |  |
| --- | --- | --- | --- |
| **Source** | **Catalogue #** | **Name** |  |
|  |  |  |  |
| All from | 115-515-146 | DyLight-594 Goat Anti-Mouse IgG | |
| Jackson ImmunoResearch | 115-484-146 | DyLight-488 Goat Anti-Mouse IgG | |
|  |  |  |  |
|  | 111-515-144 | DyLight-594 Goat Anti-Rabbit IgG | |
|  | 111-485-144 | DyLight-488 Goat Anti-Rabbit IgG | |
|  | 111-097-003 | FITC Goat Anti-Rabbit IgG | |
|  |  |  |  |
|  | 705-515-147 | Dylight-594 Donkey Anti-Goat IgG | |
|  | 705-485-147 | Dylight-488 Donkey Anti-Goat IgG | |

**List of Primers**

SABioscience RT² qPCR Primer Assay for human TH, cat # PPH02062E-200

SABioscience RT² qPCR Primer Assay for human TUBB3, cat# PPH02607A-200

SABioscience RT² qPCR Primer Assay for Human SLC6A3 (DAT), cat# PPH01449A-200

SABioscience RT² qPCR Primer Assay for Human GDNF, cat# PPH01120B-200

SABioscience RT² qPCR Primer Assay for Human KCNJ6(Girk2), cat# PPH01415A-200

SABioscience RT² qPCR Primer Assay for Human SLC18A2 (VMAT), cat# PPH01437E-200

SABioscience RT² qPCR Primer Assay for Human FOXA2, cat# PPH00976A-200

SABioscience RT² qPCR Primer Assay for Human BDNF, cat# PPH00569E-200

SABioscience RT² qPCR Primer Assay for Human NR4A2 (NURR1), cat# PPH02082A-200

SABioscience RT² qPCR Primer Assay for Human EN1, cat# PPH00986E-200

SABioscience RT² qPCR Primer Assay for Human DDC, cat# PPH19374A-200

SABioscience RT² qPCR Primer Assay for Human Pitx3, cat# PPH12380A-200

SABioscience RT² qPCR Primer Assay for Human DBH, cat# PPH02066E-200

SABioscience RT² qPCR Primer Assay for Human LMX1b, cat# PPH12240A-200

SABioscience RT² qPCR Primer Assay for Human PNMT, cat# PPH07104E-200

HKG’s : Glyceraldehyde 3-phosphate dehydrogenase (GAPDH), Tata box binding protein (TBP), succinate dehydrogenase complex subunit A (SDHA) and Glucose-6-phosphate dehydrogenase (G6PD)
